# Plasma Concentration of Platelet-Derived Microparticles Is Related to Painful Vaso-Occlusive Phenotype Severity in Sickle Cell Anemia

**DOI:** 10.1371/journal.pone.0087243

**Published:** 2014-01-24

**Authors:** Danitza Nebor, Andre Bowers, Philippe Connes, Marie-Dominique Hardy-Dessources, Jennifer Knight-Madden, Vanessa Cumming, Marvin Reid, Marc Romana

**Affiliations:** 1 Institut National de la Santé et de la Recherche Médicale, U665, Pointe-à-Pitre, Guadeloupe, France; 2 Université Antilles-Guyane, Pointe-à-Pitre, France; 3 Department of Basic Medical Sciences (Physiology Section), University of the West Indies, Kingston, Jamaica; 4 Laboratory of Excellence GR-Ex, Pointe-à-Pitre, Guadeloupe, France; 5 Laboratory ACTES, University of the French West Indies, Guadeloupe, France; 6 CAribbean network of REsearchers on Sickle cell disease and Thalassemia, Pointe-à-Pitre, Guadeloupe, France; 7 Institut Universitaire de France, Paris, France; 8 Sickle Cell Unit, Tropical Medicine Research Institute, University of the West Indies, Kingston, Jamaica; University of Torino, Italy

## Abstract

High plasma level of microparticles (MPs) deriving mainly from erythrocytes and platelets has been detected in sickle cell anemia (SCA) patients. Flow cytometry was used to determine the concentration of MPs in two groups of SCA patients exhibiting marked differences in painful vaso-occlusive crisis rates [a non-severe group (n = 17) and a severe group (n = 12)], and in a control group composed of healthy subjects (n = 20). A 3- to 4-fold increase of total MP plasma concentration was detected in SCA patients. Higher platelet-derived MPs concentration was detected in the severe SCA group while erythrocyte-derived MPs concentration was increased in the non-severe SCA patient group only. Our results suggest that plasma concentration of MPs shed by platelets is a biomarker of the vaso-occlusive phenotype-related severity.

## Introduction

Sickle cell anemia (SCA) is a hemoglobinopathy resulting from the presence of sickle hemoglobin (HbS) characterized by chronic hemolysis and recurrent vascular occlusions triggered by red blood cell (RBC) and leukocyte adhesion to the vascular endothelium [1]. Moreover, the disease is associated to a hypercoagulable and pro-inflammatory state [2] as well as endothelial dysfunction [3] and abnormal blood rheology [4], [5], [6], [7]. The clinical severity of SCA is highly variable, ranging from mild to life-threatening conditions [8].

Recently, several studies suggested that increased plasma concentration of microparticles (MPs) could be involved in the pathophysiology of SCA. Microparticles (MPs) are submicrometric fragments (0.1 to 1 mm) shed from the remodeling of plasma membrane in response to cell activation and apoptosis. They express high levels of phosphatidylserine (PS) on their outer leaflet together with surface markers from their cell of origin [9]. Elevated levels of MPs originating from circulating blood cells and endothelial cells have been reported in many vascular diseases associated with an increased risk for both arterial and venous thromboses. High plasma level of MPs has also been detected in SCA patients at steady-state [10], [11], [12] and during vaso-occlusive crisis [12]. In SCA, MPs derive mainly from erythrocytes and platelets, and to a lesser extent, from the other circulating blood cells and endothelial cells [10], [11], [12], [13]. It has been shown that erythrocytes-derived MPs isolated from SCA patients [11], [14] and MPs released by platelets exhibited pro-coagulant properties [15], which strongly suggests that these sub-cellular elements could be bio-effectors involved in the SCA pathophysiological processes.

In this study, we hypothesized that the plasma level of MPs, both total and cell-type specific, could be related to the severity of the disease defined accordingly to the rate of vaso-occlusive crises within the previous years. To test this hypothesis, we determined and compared the concentration and cellular origins of MPs in two groups of SCA patients, classified accordingly to the occurrence of painful vaso-occlusive crisis within the two years preceding their recruitment in the study and in a control group composed of healthy individuals.

## Patients and Methods

### Patients

Two groups of Jamaican SCA patients were included; a non severe group (no painful crisis within the two years preceding the recruitment; n = 17) and a severe group (at least three episodes of acute vaso-occlusive painful crises requiring day care or hospital admission and opioid analgesia within the same period; n = 12), as previously defined [7]. At the time of their inclusion, all SCA patients were in steady-state condition: i.e., no blood transfusion within the previous four months and no acute episodes (infection or vaso-occlusive crisis) for at least one month before enrolment in the study. We also studied a control group composed of healthy Jamaican individuals without hemoglobinopathy (n = 20). This study has been approved by the West Indies Ethics Committee of the Faculty of Medical Sciences/University of the West Indies/University Hospital. Written informed consent was obtained from all participants in accordance with the Declaration of Helsinki after approval of the Ethics Committee.

### Laboratory methods

Hematological parameters were obtained using an automated cell coulter (MAX M-Retic, Coulter, MIAMI, FL, USA) on EDTA blood samples. Fetal hemoglobin level was determined by high performance liquid chromatography (VARIANT ™, Bio-Rad Laboratory, Hercules, CA, USA).

### Isolation of MPs and flow cytometry analysis

Isolation and analysis of MPs by flow cytometry were performed as previously described using a FC 500 flow cytometer (Beckman Coulter) [13]. Briefly, MPs were extracted from platelet-poor plasma freshly prepared by ultracentrifugation (18,000 g, 20 min, room temperature); the pellet being washed twice in working buffer (WB; 10 mM HEPES pH 7.4, 136 mM NaCl, 5 mM KCl, 2 mM MgCl_2_) containing either 5 mM EDTA (first wash) or no EDTA (second wash). The pellet was finally suspended in WB and stored at −80°C until analysis. Extracted MPs were incubated with fluorochrome-conjugated probes, consisting of fluorescein-isothiocyanate (FITC)-annexin-V (Beckman Coulter, Brea, CA, USA) and phycoerythrin (PE)-conjugated specific monoclonal antibodies (MoAbs). The following monoclonal antibodies have been used: anti-CD15 (Lewis X, clone HI98, IgM), anti-CD41 (GPIIb, clone HI98, IgG1), anti-CD106 (VCAM1, clone 51-10 C9, IgG1), anti-CD14 (GPI, clone M5E2, IgG2a) anti-CD235a (Glycophorin A, clone 11E4B-7-6, IgG1). These monoclonal antibodies allowed the identification of the cellular origin of MPs (i.e., granulocytes, platelets, endothelial cells, monocytes and red blood cells, respectively). The following isotypic controls have been used: IgG1 (679.1Mc7), IgG2a (7T4-1F5) or IgM (G20-127). All MoAbs used in the present study were obtained from Beckman Coulter. Flow-Count™ fluorospheres (Beckman Coulter) were used for absolute MPs quantification. Flow-Count™ signal was acquired on a LogSS-LogFL3 dot-plot. The acquisition gate for MPs was standardized using the Megamix kit, a blend of size-calibrated fluorescent microbeads (0.5, 0.9 and 3 µm; Biocytex, Marseille, France), according to the supplier’s instructions.

### Statistical analysis

Differences in hematological parameters and MPs concentrations, expressed as median and range, between the various groups were assessed using non-parametric tests (Kruskal-Wallis ANOVA plus Dunn’ test for the 3 groups’ comparisons). Categorized variables such as gender and sex ratio were compared between patient groups using the chi-square test.

## Results and Discussion

As shown in Table 1, SCA patients were anemic, exhibited lower red blood cell (RBC) count and higher white blood cell (WBC) and platelet (PLT) counts than controls. No difference was detected between severe and non-severe SCA patients.

**Table 1:** Hematological characteristics of the control group and the two SCA groups.

	Controls	Non-severe SCA patients	Severe SCA patients
n	20	17	12
Age (years)	27 (21 to 43)	24 (18 to 46)	28 (22 to 46)
Sex ratio (M/F)	10/10	11/6	8/4
Hb (g/dL)	12.8 (6.6 to 15.8)	7.4 (4.7 to 11) *	7.6 (5.4 to 10.3)*
Hb F (%)	NA	6.1 (1 to 12.4)	7.1 (1.1 to 16.7)
Reticulocytes (10^9^/l)	NA	11 (2 to 17)	10 (5 to 21)
RBC (10^12^/l)	4.68 (2.67 to 6.13)	2.25 (1.74 to 5.38) *	2.55 (1.53 to 3.49) *
Platelets (10^9^/l)	238 (170 to 750)	368 (196 to 1048) *	391 (186 to 608)*
WBC (10^9^/l)	5.3 (4.1 to 12.4)	11 (6.7 to 15) *	10.5 (4.1 to 16.4)*

All values are expressed as median and ranges. RBC: red blood cells; WBC: white blood cells; NA: not available. * different from controls (p< 0.05)

**Figure 1:**
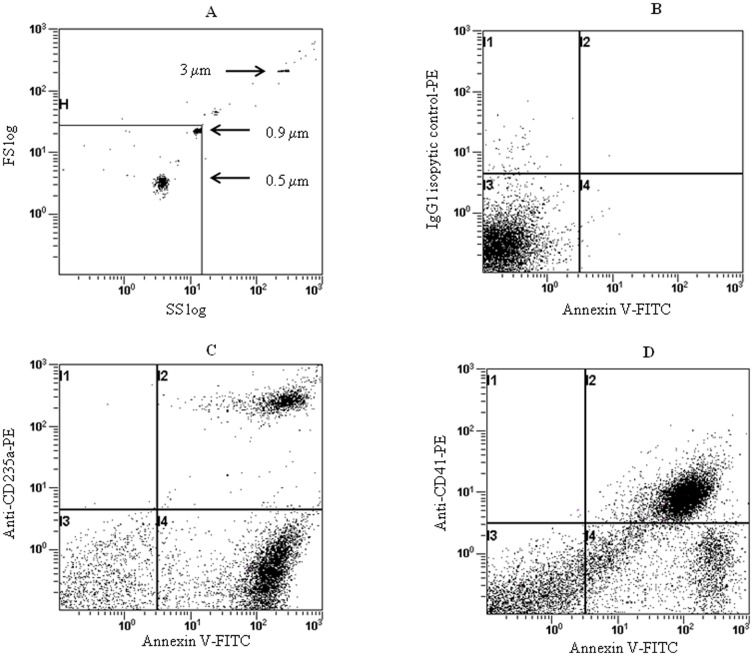
Representative flow cytometry analysis for quantifying microparticles originated from red blood cells and platelets. Figure 1A: Acquisition gate set up using the fluorescent microbeads size of the megamix kit according to the manufacturer instructions. Figure 1B: negative control using isotypic control-PE and annexin V-FITC in EDTA buffer. Figure 1C: double fluorescence plots demonstrating MPs originated from RBCs. Figure 1D. double fluorescence plots demonstrating MPs originated from platelets.

**Figure 2:**
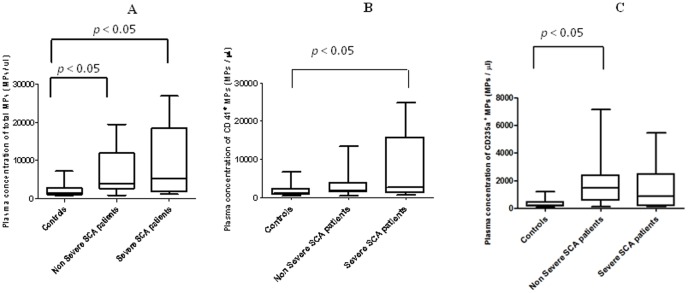
Comparison of MPs concentration between control group and SCA patients groups. Figure 2A: Total MPs. Figure 2B: Platelet-derived MPs. Figure 2C: Erythrocyte-derived MPs.

In order to quantify and identify MPs released by RBCs, PLTs, monocytes, granulocytes and endothelial cells, we used a two color flow cytometry assay with Annexin V-PE and MoAb-FITC specific of each of these blood cell types. Representative flow cytometry fluorescence dot plots for quantifying RBC- and PLT-derived MPs (i.e. the most frequently detected MPs in the plasma of both controls and SCA patients) are shown in Figure 1. A 3 to 4 fold increase of total MP plasma concentration was detected in SCA patients compared to control subjects (4,149 [857 to 26,863] vs 1,410 [696 to 7,158] respectively, *p* = 0.0001) as previously reported [11], [13]. We then compared the concentration of total MPs and cell-type specific derived MPs between controls and SCA patients classified according to the disease severity. Higher total MPs concentrations were detected in the severe and non-severe SCA groups (Figure 2A). Compared to control individuals, greater PLT-derived MPs concentration was detected in the severe SCA group while the non-severe SCA group exhibited intermediate level (Figure 2B). In contrast, RBC-derived MPs concentration was significantly increased in the non-severe SCA patient group only (Figure 2C). No difference was observed between the three groups for MPs originated from monocytes, granulocytes and endothelial cells, which were detected at very low levels (data not shown).

To our best knowledge, the relationship between plasma PLT-derived MPs level and vaso-occlusive phenotype-related severity described in the present study has never been reported so far. The higher level of PLT-derived MPs concentration in the severe SCA group compared to the control group was not related to the two known conditions affecting the plasma level of MPs in SCA patients: 1) none of our patients was treated by hydroxycarbamide, a condition known to modulate both total MP and PLT-derived MP levels in SCA patients [13], [16], and 2) fetal hemoglobin level, which has been reported to be inversely correlated with the plasma concentration of MPs [13], was not different between non-severe and severe SCA patients (6.1 % vs. 7.1 %, *p* = 0.34, respectively) and was not correlated with PLT-derived MPs (rho  =  -0.24, *p* = 0.22). Moreover, it seems unlikely that this higher MP concentration could be the direct consequence of previous acute vaso-occlusive events. Indeed, SCA patients included were in steady state condition and it has been previously shown that these sub-cellular elements are rapidly removed from the circulation [17], [18]. Previous data suggested that plasma MPs could promote thrombotic-like events in SCA and participate to the pathophysiology of the disease [19], [20]. Circulating PLT-derived MPs have been shown to express high level of tissue factor and phosphaditylserine at the membrane surface; both factors that may promote and thrombin generation and coagulation [9], [21], [22]. Indeed, it is tempting to hypothesize that PLT-derived MPs reflect the severity of the painful vaso-occlusive phenotype.

The quantitative pattern of RBC-derived MPs is more puzzling with the highest plasma concentration detected in the mild SCA patient group. This result contrasts with a recent study showing that RBC-derived MPs compromised vasodilatation in kidney microcirculation, induced *in vitro* production of radical oxygen species by endothelial cells and triggered kidney vascular occlusion in transgenic SAD mice [23]. Unfortunately, kidney function was not assessed in this study, leading unresolved the relationship between RBC-derived MPs and renal function in humans with SCA. A recent study had provided evidence that SCA patients with high RBC-derived MPs plasma level exhibited high hemolytic rate [24]. However, painful vaso-occlusive crisis is not a complication resulting from chronic high hemolytic rate [25]. Instead, this complication results from low hemolytic rate and increased blood viscosity, which may compromise tissue perfusion [6], [7]. Complications from the hemolytic-endothelial dysfunction phenotype, such as pulmonary hypertension, glomerulopathy, stroke, leg ulcers or priapism [25], have not been investigated in the present study and we ignore whether the mild SCA group had higher rate for these complications. Clearly, the number of patients included in the present study does not allow us to analyze such association and further studies are warranted to clarify the impact of these MPs on the SCA phenotype.

The unexpected qualitative pattern of MPs detected in the severe group should be reproduced in other SCA cohorts to strengthen this finding. Furthermore, although RBC- and PLT-derived MPs in SCA patients are known to exhibit a pro-inflammatory, pro-coagulant and pro-adherent phenotype [2], [3], their impact on the biology of circulating blood cells and endothelial cells is poorly known and needs to be further explored.

In summary, our results suggest that plasma concentration of MPs shed by platelets is a biomarker of the vaso-occlusive phenotype-related severity. Larger studies focusing on the relationship between plasma concentrations of these sub-cellular elements and the other SCA clinical manifestation should be undertaken. In addition, larger cohort studies are needed to better understand the physiological meaning of the wide inter-individual variability of MPs concentration in SCA patients.
